# Efficacy of Electroconvulsive Therapy in Bipolar Disorder with Mixed Features

**DOI:** 10.1155/2016/8306071

**Published:** 2016-01-05

**Authors:** Miguel Palma, Berta Ferreira, Nuno Borja-Santos, Bruno Trancas, Céu Monteiro, Graça Cardoso

**Affiliations:** ^1^Department of Psychiatry, Hospital Prof. Doutor Fernando Fonseca, EPE, 2720-276 Amadora, Portugal; ^2^Day-Hospital, Lisbon Psychiatric Hospital Center, 1749-002 Lisbon, Portugal; ^3^Chronic Diseases Research Center (CEDOC) and Department of Mental Health, NOVA Medical School, New University of Lisbon, 1150-082 Lisbon, Portugal

## Abstract

*Introduction.* Mixed states represent a frequent presentation of bipolar disorder, associated with higher resistance to psychopharmacology. Limited evidence supports the use of ECT in these patients. We aim to report our experience on treating bipolar mixed states with ECT.* Methods.* Retrospective data were collected from all bipolar patients submitted to acute ECT treatment, between June 2006 and June 2011. Three groups were created in terms of affective polarity of the episode. CGI rating was used to establish clinical remission and demographic and clinical variables were compared among groups. Long-term outcome was assessed through readmission measures, considering the use of continuation or maintenance ECT.* Results.* During the study time frame, a total of 50 ECT course treatments were performed on 41 bipolar patients. All affective episodes, except one mixed state, showed a positive clinical response. Patients with mixed state presentation tended to be younger and have an earlier first hospitalization than depressed patients. No differences were found in terms of ECT sessions performed, length of hospital admission, referral to continuation ECT treatment, number of readmissions, and time until next readmission.* Conclusions.* Our results support the effectiveness of ECT in patients experiencing a mixed affective state.

## 1. Introduction

Bipolar mixed states were systematically described for the first time by Kraepelin [1]. Since then, their high prevalence has been repeatedly recognized, occurring in as many as 50% of all patients with affective bipolar disorders [2], but they still remain poorly understood. Mixed states are known to be associated with worse prognosis than depressive or manic forms of bipolar disorder [3]. Comorbidities such as substance abuse, traumatic brain injury, and other neuropsychiatric and brain development impairments seem to facilitate the emergence of mixed states [4]. Indeed, the risk for developing mixed affective states appears to be nowadays increased in part due to widespread use of antidepressants [5] and substance abuse [6], and its occurrence might be substantially underestimated by too restrictive classificatory criteria. During the period that ranged from DSM-III to DSM-IV-TR, in order to be diagnosed with a mixed state, patients had to experience concurrent full depressive and manic syndromes for more than one week, leaving aside the majority of cases where subsyndromal mixed symptoms were present [7–10]. The same still happens today under the aegis of ICD-10 [11]. Some other unofficial classifications were created that comprised broader criteria to diagnose mixed state entities [12–14]. They generally require a minimum of two or three symptoms of opposed polarity within a major affective episode. Clinical validity and utility of entities such as dysphoric mania or hypomania, depression with flight of ideas, or excited depression have been substantially confirmed on empirical grounds [12, 14].

Lately, DSM-5 acknowledged and corrected this gap, introducing mixed features as a specifier of manic, hypomanic, and major depressive episodes in bipolar I or bipolar II disorder or major depressive disorder [15]. This change of practice, though late and controversial [16], may result in a more prompt recognition of underlying bipolar diathesis in depressed patients where no past history of manic/hypomanic episodes was identifiable, therefore allowing a more suitable treatment, especially demanding a cautious use of antidepressants.

Furthermore, the consequent increase in prevalence of mixed states requires a better understanding of risk factors, treatment, and prognosis of these patients. As a matter of fact, they are usually extremely difficult to treat, many being refractory to psychotropic drugs [17]. In comparison with depressed patients, those afflicted with mixed states appear to have longer and more severe episodes, higher frequency of psychotic features, lower interepisodic remission, and greater risk of suicide [18, 19]. Due to all these dissimilarities, mixed states surely deserve to be analyzed per se, rather than just being bundled within studies primarily focused on depression or mania. In line with scientific evidence on the effectiveness of Electroconvulsive Therapy (ECT) in the treatment of both depressed and manic phases of bipolar disorders, clinical experience also supports its use in mixed states. Interestingly, in 1938, Cerletti and Bini departed from animal studies to the first ever human trial of ECT in a patient allegedly suffering from a mixed state episode with psychotic symptoms [20]. There is, however, little information in the scientific literature, supporting ECT to be equally efficacious in these patients. Small et al. were among the first to suggest ECT as a satisfactory approach in mixed states after concluding that manic patients with concomitant depressive symptoms showed larger improvements with ECT [21]. Since then, five studies have been published on the efficacy of ECT treatment in mixed state patients [22–26]; two of them compared results to depressed patients [24, 25] and only one to both depressed and manic patients [23]. Our study aims to report our experience in using acute phase ECT (aECT) for the purpose of relieving acute symptoms in patients with mixed affective states that previously failed to respond to pharmacotherapy, therefore adding scientific evidence to this underexplored field of knowledge. Moreover, we analyse our results with regard to the follow-up of these patients and the outcomes of application of pharmacotherapy alone or in conjunction with continuation (cECT) and maintenance ECT (mECT).

## 2. Methods

### 2.1. Patient Selection

We retrospectively collected clinical data from all patients diagnosed with bipolar disorder treated with aECT, in our unit (Department of Psychiatry of Hospital Prof. Doutor Fernando Fonseca, EPE), between June 2006 and June 2011. Patients included were at least 18 years old and treatment courses had to comprise a minimum of 2 sessions of acute regularity and no interruption due to medical reasons. Consideration for ECT treatment was clinically based, and drug resistance remained the main criteria. All patients included in the study failed to show consistent response to psychopharmacology, although no formal assessment of treatment resistance had been performed.

### 2.2. Data Collection

During the study period, some patients presented more than one mood episode requiring aECT treatment. As our main objective was to compare efficacy of ECT on resolution of acute symptoms, an episode-based approach was pursued. Bipolar affective episodes were split up in three different groups according to its polarity and evaluated in terms of clinical and treatment characteristics (electrode placement, number of ECT sessions per course, length of hospitalization, number of readmissions, and time to readmission). For matters of clarity, a patient-based comparison of clinical and demographic features was performed. Patients were classified by the affective polarity of the index episode. After resolution of acute symptoms with index treatment, some episodes were kept on medication alone whereas in others cECT (up to 6 months after aECT) was added to prevent relapse. Among the latter, recurrence prevention of new mood episodes after the end of cECT was attained with medication alone or in combination with mECT. In a secondary analysis of outcome, readmission rate and time to readmission were compared between episodes with either choice of treatment. Diagnoses of depressive and manic episode were made when the patient met DSM-IV-TR criteria [10], while mixed states were diagnosed according to McElroy's (mania/hypomania episode plus 3 or more depressive symptoms) [12] and Akiskal's criteria (major depressive episode plus 2-3 manic/hypomanic symptoms) [14]. Retrospective rating for severity of illness was made using Clinical Global Impression (CGI), and a CGI-S score equal to 3 (mildly ill) or less at the end of an aECT course was adopted as a positive clinical response criterion.

### 2.3. ECT Treatment

Written informed consent for ECT was obtained and treatments were administered twice a week. At admission, all the patients were receiving psychotropic medications, depending on physician's choice, namely, antipsychotics and mood stabilizers, though the latter as well as benzodiazepines had been regularly interrupted the night before each treatment, to minimize interference on the seizure threshold or lithium neurotoxicity. Treatments were performed with a Somatics Thymatron System IV Brief Pulse device. Frequency (10–140 Hz), pulse width (0.25–1.5 ms), duration (0.14–8.0 s), and current (0.9 A constant) measures were automatically calculated, whereas energy (5–199.6 J) was set according to dose-titration method using the recommendations by the manufacturer [27]. Due to a change in the internal protocol, ECT had been administered with either bitemporal or bifrontal electrode placement, according to the current practice at the time of treatment. Propofol and etomidate (doses dependent on weight and previous response) were used as anaesthetic inducers, followed by the muscle relaxant succinylcholine (0.5–1 mg/kg). Patients were ventilated on 100% O_2_ until resumption of spontaneous respiration. Length of the generalized seizure elicited (≥15 seconds by motor criteria) was used as a measure of adequacy of treatment. Motor response was monitored using the cuff technique and EEG was also recorded. In case of missed or inadequate seizure, the patient was restimulated in increasing steps of intensity up to a maximum of four stimulations in a row, according to the same dose-titration schedule, after which titration was postponed to a following session [27]. Acute ECT courses were terminated when the physician in charge considered that the aimed therapeutic benefits were achieved or whenever a sustainable appropriate response was followed by a clinical plateau over two consecutive stimulations or in case of unsatisfactory or absent symptomatic improvement observed after, at least, four consecutive adequate seizures with intensified stimuli. On follow-up, frequency of sessions ranged from weekly to every 3 weeks in cECT and from fortnightly to monthly in mECT.

### 2.4. Statistical Analysis

Because assumption of normality in data could not be made, we had to rely on nonparametric statistical tests. Comparisons between three groups were performed using Chi-square analysis (or Freeman-Halton extension of the Fisher Exact Probability test, when appropriate) for categorical variables and Kruskal-Wallis test for continuous variables. Comparisons between two independent groups were performed using Chi-square analysis (or Fisher Exact Probability test) for categorical variables and Mann Whitney test for continuous variables. The significance level for each test was established at *p* < 0.05, 2-tailed. All statistical analyses were performed using Statistical Package for Social Sciences (SPSS-20.0, IBM Corporation, Armonk, NY), except for the Freeman-Halton extension of the Fisher Exact Probability test where a website for statistical computation was used (http://vassarstats.net/).

## 3. Results

Forty-four consecutive patients met inclusion criteria, but 3 were excluded due to incomplete information. Of the 41 patients that comprised our sample, 30 were female (73.2%) and 11 were male (26.8%), while for ethnicity 30 were white (73.2%) and 11 were black (26.8%). A total of 50 course treatments were performed during the period of the study. Interestingly, all the 9 patients included that had more than one affective episode requiring ECT treatment had relapses that were in the very same affective polarity. In particular, four patients presented with two depressive consecutive episodes and one patient with two mixed episodes demanding ECT treatment. Two other patients had three relapses treated with courses of ECT (one with three depressive episodes and the other with three mixed episodes).

Overall, the mixed state group represented 36.6% (*n* = 15), the depressed group 53.7% (*n* = 22), and the manic group 9.8% (*n* = 4) of the total population of patients.  Table 1 presents the demographic and clinical features of these 3 subpopulations.

Due to the small size of the manic population, performing comparisons was hindered. Therefore we repeated the comparisons, excluding the manic group. Because normality was not observed in any of the variables for depressed and mixed state population, nonparametric tests were used. Patients diagnosed with mixed states tended to be younger than depressed patients at the time of the study (*p* = 0.063) and at first admission (*p* = 0.057), but no differences were observed in the number of previous admissions (*p* = 0.157). Both depressive and mixed state groups had a higher percentage of female patients and no differences in gender distributions were found (*p* = 1.000).

Considering the total number of 50 episodes that demanded ECT treatment, psychotic symptoms were present in 42.9% of depressive episodes and in 44.4% of mixed state group. Two manic patients were psychotic while the other 2 were not (*p* = 0.999).

Due to a modification in the internal protocol that rules our technical practice for applying ECT, a variable proportion of patients within each group had one of two different electrode placements. In the depressed group, 20 cases (71.4%) had bitemporal electrodes, while 8 (28.6%) had bifrontal. In the mixed group, in 8 (44.4%) of the cases electrodes were bitemporally placed, and in 10 (55.6%) bifrontally. All the four manic patients received bifrontal placement (*p* = 0.012). Differences between depressed and mixed group were nonsignificant (*p* = 0.067).

Chart review of episodes' descriptions at the moment of referral to the ECT unit indicated moderate to severe mental illness. All treatments but one in a mixed state patient showed positive clinical response, as documented on retrospective rating of CGI equal or inferior to 3.

We also compared whether any relevant difference could be found between groups in terms of number of ECT sessions performed and length of hospital admission. These represent indirect measures of treatment efficacy and constitute relevant dimensions of care concerning the cost-benefit of this treatment. The average number of ECT sessions in the mixed state group was slightly lower in mixed state episodes than manic or depressive episodes, although not reaching statistical significance. When considering the days of hospitalization during the affective episode that motivated ECT treatment, manic episodes were associated with longer admissions than mixed states or depressive episodes (Table 2). No difference arose from a direct comparison between episodes of depression and mixed states (*p* = 0.955).

After the acute treatment, patients were put on psychopharmacologic medication alone or augmented with cECT. All patients in our sample were either on mood stabilizer or antipsychotic medication, and in two thirds with a combination of both. In the depressed group, 20 episodes were put on mood stabilizers, 22 on antipsychotics, and 5 on antidepressants (never in monotherapy). In what concerns mood stabilizers, lamotrigine was the drug of choice in 12 depressive episodes while 5 were put on lithium and 3 on sodium valproate; lamotrigine was associated thrice with lithium and once with sodium valproate. Only one patient with a depressed episode was put on long-acting antipsychotic; four were on SSRI and one on SNRI. In the mixed state group, 11 episodes were medicated with mood stabilizers (lithium and lamotrigine in 5 situations each, 3 of which with both drugs combined; 4 with sodium valproate), 13 were put on antipsychotic (of which 6 were on long-acting medication), and no antidepressant was prescribed. Finally, in the manic group, all 4 episodes were on combination of mood stabilizer (3 with sodium valproate and 2 with lithium) and antipsychotic (2 with long-acting drugs), and none on antidepressant.

The choice for the combined approach of medication plus cECT was carried out in slightly more than one-third of depressive episodes, while in the manic and mixed state groups this decision was taken in more than two-thirds of cases (Table 2). Direct comparison between depressive and mixed state episodes revealed no statistical difference (*p* = 0.132). Eight out of 10 (80.0%) of the depressive episodes that started cECT were kept on maintenance regime, while the proportion in mixed state group was of 7 out of 11 (63.6%). Only 1 manic episode (33.3%) kept taking ECT after the 6 months of continuation phase. No differences were observed in a 3-group comparison analysis (*p* = 0.362) or when excluding the manic group (*p* = 0.635).

The mean duration of follow-up was 136.4 ± 80.1 weeks (range: 10.1–249.9 weeks), and no significant differences were observed between depressed and mixed state groups (depressed group = 149.0 ± SD 86.0 versus mixed group = 125.7 ± SD 76.4 weeks, *p* = 0.344). Although the mixed state group presented a higher percentage of episodes followed by readmission due to affective relapse in comparison to the depressive group, the absolute number of readmissions that followed the index admission did not differ between both groups (average number of readmissions: depressed group = 0.6 ± SD 1.3 versus mixed group = 0.9 ± SD 1.3; *p* = 0.353), neither did the average time to readmission (mixed readmitted patients = 48.3 ± SD 62.0 versus depressed readmitted patients = 47.3 ± SD 48.3 weeks; *p* = 0.424).

Further analysis, irrespective of mood polarity, revealed no differences in readmission rate in terms of selection or not for cECT. One-third of mood episodes (8 in 24) referred for cECT had to be readmitted during follow-up; however, only 3 hospitalizations occurred while on this treatment. Although no significant differences were observed in terms of mean number of readmissions or time until next hospitalization, readmission of episodes treated with cECT occurred later (on average), albeit a high variability of this measure. Moreover, the elevated number of readmissions in the c-ECT group indicates a highly severe sample of patients (Table 3). Among the group of episodes treated with cECT, 16 proceeded to maintenance modality (depressed group = 8, manic group = 1, mixed state group = 7), while 8 did not. A total of 11 readmissions occurred under mECT (versus 1 readmitted patient in the no-mECT group) but 5 of those relapses took place in the very same patient (depressed group). The percentage of admissions that occurred within a period of time coincidental with ECT treatment (on either continuation or maintenance regime) was equal in depressed and mixed state group (7 out of 17 readmissions, 41.2%).

## 4. Discussion

This paper is intended as a reflection about the practice of our department in the treatment of patients with bipolar disorder, especially in what concerns their referral to the ECT unit. Moreover, our customary practice of referring mixed state patients to be treated with ECT called for a systematic analysis of these data, in order to add it to the little evidence available about the efficacy of ECT in the treatment of bipolar episodes with mixed features. Our results support the validity of ECT as an effective treatment for mixed bipolar episodes, comparable to the effect on relapses in depressive and manic polarities. All the patients included in the study, except one in the mixed group, showed a positive response to aECT course. The number of ECT treatments did not differ as a function of patient diagnostic group; however, we could even appreciate a trend towards shorter course treatments in the mixed group. In part due to the appreciation of adequacy of ECT treatment on mixed state patients, three quarters of the episodes with acute treatment remained on maintenance ECT. However, half of the episodes were followed by the need for readmission, although almost half of these patients were in maintenance treatment. These results, although higher in mixed episodes, did not significantly differ from the depressed group. The massive response rate across all polarities prevented the finding of predictors of response.

As far as we know, only a disappointing number of five studies had been published on the efficacy of ECT treatment in patients with bipolar mixed states [22–26]. Gruber et al. reported a series of 7 mixed state cases submitted to ECT, stating that remission criteria were achieved in all patients and both depressive and manic symptoms significantly improved over ECT trials [22]. Devanand et al. also showed overlapping benefits of ECT treatments over the three polarities of bipolar episodes (depressed, *n* = 38; manic, *n* = 5; mixed, *n* = 10), even if mixed patients required longer hospitalizations and aECT courses [23]. A study by Ciapparelli et al. did not find any difference between treatment-resistant bipolar patients with depression (*n* = 23) and mixed manic episodes (*n* = 41) in terms of number of aECT sessions needed, although mixed manic patients had a higher proportion of responders at endpoint (26% versus 56%) [24]. Medda et al. also compared bipolar depressed (*n* = 46) and mixed state patients (*n* = 50) and observed a similar number of ECT sessions between groups (7.4 ± SD 2.5 versus 7.4 ± SD 2.4) and a more balanced response rate (67.4% versus 76.0%) [25]. The same group published another study, focusing only on mixed state patients treated with ECT. Response was achieved in 72.1% of the sample. Several clinical and technical variables failed to show association with quality of response. Nonresponders (27.9% of total sample) had longer duration of the current mixed episode. Remitters (30.5%) had less frequent OCD lifetime comorbidity and presented at baseline with higher depressive symptoms but lower manic features than responders and nonresponders [26]. All these mentioned studies used DSM-IV or Research Domain Criteria to diagnose mixed state, differing in terms of requirements to qualify for response.

Due to the strong community-based model in which our department is set up, patients with bipolar disorder are assertively followed up by a mental health team in the community. Whenever patients present with an affective relapse, they are primarily treated as outpatients up to the moment that severity of illness or lack of social support requires admission to the inpatient unit. ECT remains as a therapeutic resource to both outpatients and inpatients and is considered whenever psychopharmacologic failure has been detected. However, aECT treatment is usually taken as first therapeutic strategy for patients that have been successfully treated by that approach in previous affective episodes. In fact, 9 out of the 50 episodes of aECT treatments analysed refer to patients that had been adequately treated before. In these cases ECT was proposed as a first-line treatment and the patient consented to it. Our analysis by episode had the limitation of considering each episode as independent when in fact that was not the case in almost one-fifth of them. Repeating the analysis excluding all the acute treatments in previously treated patients did not change, however, any of the reported results.

The retrospective and naturalistic set-up of this study includes some limitations that we are aware of. Measures of global improvement were attributed by chart review and concomitant medication interferes with our aim to isolate ECT therapeutic effect on bipolar mood episodes. Our higher than normal response rate can be partially explained by the retrospective and quite broad concept of response (CGI-S ≤ 3), but also with a loser criterion of treatment resistance that was not subject to formal assessment; our study sample is therefore likely to comprise drug-responsive patients, had the pharmacological approach been pursued in a longer and more aggressive fashion. Additionally, a change of practice in electrode placement (from bitemporal to bifrontal) could have introduced a confounding variable that we did not control for. However, the literature suggests that the clinical differences between either of these bilateral techniques seem to be modest, if any, and may rest upon slightly faster response in bitemporal position and probably less cognitive effects in bifrontal position, which was the rational for changing our protocol [28–30]. The fact that more mixed patients had bifrontal electrodes is likely a reflex of our increasing referral of these patients for ECT treatment, since this is the current established protocol in our unit. This late recognition of mixed episodes as privileged candidates for ECT could have shortened the follow-up time reducing the chances for relapse or readmission. Having said that, analysis of the duration of time since discharge to follow-up endpoint (June 2011), although slightly longer in depressed episodes, did not significantly differ from mixed state ones. Careful analyses of patients' records did not report any relevant adverse event during ECT sessions, and this comes in accordance with the notion of high safety and tolerability of the procedure in patients with bipolar disorder [31]. Unfortunately, cognitive measures were not systematically assessed in our study. The small sample size is another major limitation. It is interesting to highlight the low number of manic patients referred for treatment with ECT. Several reasons concur to this fact. First of all, the broadly inclusive criteria for the diagnosis of mixed patients, ranging from excited depression to dysphoric mania, have as a consequence a decreasing number of patients diagnosed with depressive or manic episodes. Therefore, only pure depression and mania remained with this diagnostic category. Because we require previous informed consent from the patient when referring to ECT, a significant amount of manic patients would have refused to be submitted to ECT due to their lack of insight and grandiose psychopathologic experiences. Finally, a superior arsenal of pharmacologic choices is available to patients with mania, which implies that successful treatments could have prevented considering ECT as an alternative.

Despite these limitations, our sample had the advantage to consider a broader concept of mixed states, joining the current diagnostic trend of considering depression and mania only in their most pure presentations. As far as we know, this is the third study approaching the efficacy of ECT in mixed state patients compared to other polarities of illness and although these other papers did not report their data on follow-up length, this is most likely one with the longest. In fact, very little is known about the impact of aECT on the long-term outcome of bipolar patients. Medda et al. recently published a prospective naturalistic study that followed 36 bipolar patients with a medication-resistant severe depression or mixed state index episode that responded to aECT (mean duration = 55.3 ± SD 30.4 weeks). Of these, 13 had a depressive relapse, 5 months on average after the end of acute treatment; one patient had a mixed state relapse [32]. Concordantly, among our episodes that were not referred for cECT, half of readmissions occurred in the 15 weeks following termination of aECT, supporting preliminary evidence that the benefits of this treatment in bipolar patients tend to vanish within the first months after last treatment [32].

Furthermore, although cECT and mECT have been suggested, very limited data has been published so far [33, 34]. It is expected that the ongoing PRIDE study from the CORE Group might bring some additional new evidence [35]. Our results add, therefore, to the available evidence that mixed state episodes are, at least, as responsive to aECT as depressive and manic phases, suggesting that this treatment is adequate to bipolar illness, regardless of the form of affective relapse. Because mixed states are especially refractory to conventional psychopharmacological approaches, ECT should be considered as a valid alternative in the treatment of these patients.

## Figures and Tables

**Table 1:** Demographic and clinical characteristics of the sample of patients (*n* = 41).

	Bipolar depressed patients	Bipolar manic patients	Bipolar mixed patients	*p* value
	(*n* = 22)	(*n* = 4)	(*n* = 15)	
Females, *n* (%)	17 (77.3%)	2 (50%)	11 (73.3%)	0.528^a^

Age, in years, on 31 July 2011, mean ± SD	57.9 ± 19.0	42.9 ± 13.1	45.0 ± 15.5	0.089^b^

Age, in years, at 1st admission due to affective episode, mean ± SD	51.4 ± 19.4	32.8 ± 13.8	38.9 ± 16.0	0.061^c^

Number of previous admissions, mean ± SD	1.8 ± 2.7	4.0 ± 1.4	2.7 ± 2.4	0.057^d^

^a^Freeman-Halton extension of the Fisher Exact Probability test; ^b^Kruskal Wallis test, *χ* ^2^ = 4.834, df = 2; ^c^Kruskal Wallis test, *χ* ^2^ = 5.605, df = 2; ^d^Kruskal Wallis test, *χ* ^2^ = 5.747, df = 2.

**Table 2:** Clinical characteristics of the sample of episodes (*n* = 50).

	Bipolar depressed episodes	Bipolar manic episodes	Bipolar mixed episodes	*p* value
	(*n* = 28)	(*n* = 4)	(*n* = 18)	
Number of ECT sessions, mean ± SD	5.9 ± 3.2	6.8 ± 3.3	5.1 ± 2.2	0.336^e^

Length of hospitalization during the episode, in days, mean ± SD	39.2 ± 30.2	57.0 ± 22.3	44.7 ± 54.5	0.250^f^

Continuation ECT, *n* (%)	10 (35.7%)	3 (75.0%)	11 (61.1%)	0.174^g^

Readmission due to affective relapse, *n* (%)	10 (35.7%)	0 (0%)	8 (44.4%)	0.179^g^

^e^Kruskal-Wallis test, *χ* ^2^ = 2.180, df = 2; ^f^Kruskal-Wallis test, *χ* ^2^ = 2.770, df = 2; ^g^Freeman-Halton extension of the Fisher Exact Probability.

**Table 3:** Readmission characteristics of episodes that were treated or not treated with cECT (*n* = 50).

Continuation ECT	Yes	No	*p* value
	(*n* = 24)	(*n* = 26)	
Readmission due to affective relapse, *n* (%)	8 (33.3%)	10 (38.5%)	0.920^h^
Time to readmission, in weeks, mean ± SD	73.6 ± 65.4	27.1 ± 30.5	0.248^i^
Total number of readmissions, *n*; mean ± SD	17; 0.71 ± 1.459	11; 0.65 ± 1.018	0.741^i^

^h^Chi-square; ^i^Mann-Whitney *U* test.
